# A review of the evidence on cigarettes with reduced addictiveness potential

**DOI:** 10.1016/j.drugpo.2021.103436

**Published:** 2022-01

**Authors:** Eric C. Donny, Cassidy M. White

**Affiliations:** Wake Forest University School of Medicine, Department of Physiology and Pharmacology, Winston-Salem, NC, USA

**Keywords:** Smoking, Tobacco control, Nicotine regulation, Cigarettes, Addiction, Cessation

## Abstract

**Background:**

In May 2018, the Secretariat for the World Health Organization Framework Convention on Tobacco Control convened a meeting to discuss the potential for reducing the addictiveness of tobacco products. A central focus was to review research findings on the behavioral effects of reducing the addictiveness of cigarettes.

**Methods:**

This manuscript reports the results of a review of the behavioral science literature, updated through April 2021, with special attention to both the potential benefits and unintended consequences of reducing nicotine in cigarettes.

**Results:**

Available evidence suggests that reducing nicotine content in cigarettes to very low levels could benefit public health in three primary ways, by 1) decreasing uptake of regular smoking, 2) decreasing the amount people smoke, and 3) increasing the likelihood of smoking cessation. Current evidence also suggests that reducing nicotine in cigarettes may produce similar benefits across many important subpopulations of people who smoke, including those with psychiatric comorbidities, those who use other substances, those with low socioeconomic status, young people, people who smoke infrequently and people who prefer menthol cigarettes. Cigarette nicotine reduction could also lead to some undesirable outcomes, such as experiencing withdrawal, product manipulation, an illicit market, and harm misperceptions; strategies that may mitigate each are discussed.

**Conclusion:**

Overall, behavioral research suggests product standards that limit the nicotine content of combusted tobacco products could render cigarettes and similar products less addictive. The availability of legal, non-combusted products that effectively substitute for cigarettes and the dissemination of public health campaigns that clarify misperceptions about the relationship between nicotine, tobacco and disease may facilitate the extent to which a nicotine reduction policy reduces smoking.

## Introduction

Over a billion people worldwide smoke cigarettes (Office of the Surgeon General, 2014; Shafey, 2009) and a third to a half of lifetime smokers will die from tobacco-related illnesses (Doll, Peto, Boreham, & Sutherland, 2004; Fagerstrom, 2002; Jha, 2009). While tobacco control policies have effectively reduced tobacco use, the implementation and impact of existing policies have been heterogeneous and smoking remains prevalent in many parts of the world (World Health Organization, 2015).

To date, few policies directly target a key, underlying cause of chronic cigarette use – nicotine reinforcement and dependence. Product standards that set a maximum nicotine level are receiving attention as possible extensions to existing tobacco control efforts. The U.S. is currently considering such a policy, with the Food and Drug Administration releasing an Advanced Notice of Proposed Rule Making in 2018 (Gottlieb & Zeller, 2017; U.S. Food & Drug Administration, 2018). Similar policy action is being discussed elsewhere in the world (Donny, Walker, Hatsukami, & Bullen, 2017; Health Canada, 2017; New Zealand Ministry of Health, 2021; Proctor, 2011; World Health Organization, 2019). In particular, Article 9 of the World Health Organization (WHO) Framework Convention on Tobacco Control (FCTC) encourages parties to this treaty to establish “guidelines for testing and measuring the contents and emissions of tobacco products, and for the regulation of these contents and emissions” (World Health Organization, 2005, p.9). Following the 7th Conference of the Parties to the WHO FCTC, the Convention Secretariat organized a meeting to discuss methods for reducing the addictiveness of tobacco products. This meeting convened in May 2018 in Berlin, Germany and highlighted research that has been done to anticipate the effects of nicotine reduction product standards (WHO, 2018).

While the impact of any given policy cannot be fully determined before implementation, behavioral research can offer important insight to policy analysts and policy makers about how behavior may change when cigarette nicotine content is reduced. The objective of this paper is to provide an updated overview of the behavioral science relevant to anticipating the potential effects of reduced nicotine cigarettes for countries considering policies aligned with Article 9 of the WHO FCTC, “Regulation of the contents of tobacco products.” The paper assumes the goal of such a policy would be to reduce the death and disease caused by smoked tobacco. Hence, we approach this policy as a form of harm reduction in which regulations target the legal manufacturing, distribution, and commercial sale of combusted products with the intent of shifting how people use nicotine (to less toxic products) rather than to prohibit nicotine use *per se*.

Cigarettes were engineered over decades to maximize the delivery of nicotine and the consequent pharmacological actions that drive addiction (Centers for Disease Control, 2010; Office of the Surgeon General, 2014). Other constituents (e.g., acetaldehyde), design features (e.g., ventilation), and non-pharmacological factors (e.g., marketing, cost) may also impact use (Carter et al., 2009). However, decades of research suggest that nicotine is the primary determinant of cigarette addiction (Centers for Disease Control, 2010; Office of the Surgeon General, 2014). We focus on cigarettes, as most relevant research assesses the impact of reformulation in people who smoke cigarettes.

Long-term behavior change is the key outcome of any strategy for reducing addictiveness. Low nicotine cigarettes are still highly toxic; indeed, they reduce the ratio of the drug nicotine (which is relatively safe) to byproducts of combustion (which are not safe). On the face, this approach appears to be inconsistent with harm reduction. However, reducing the harm caused by nicotine is ultimately about shifting behavior away from smoking. Therefore, the impact of low nicotine cigarettes on public health will largely be a function of how effectively they reduce use of combusted tobacco (not whether they reduce use of nicotine). Consequently, behavioral outcomes reviewed herein include the uptake of regular smoking, the intensity of smoking, and smoking cessation.

This paper builds on previous reviews of nicotine reduction research (Berman & Glasser, 2019; Donny et al., 2014; Hatsukami, Perkins et al., 2010; Smith, Rupprecht et al., 2017; Stanton & Hatsukami, 2019), adding studies published through April 2021. We also extend these reviews by summarizing the available evidence on reduced uptake, as an additional category of behavioral response relative to public health impact. Finally, we review evidence related to potential negative consequences, including withdrawal among people who currently smoke, misperceptions of reduced harm, and the manipulation of products.

## Methods

An initial review of the behavioral evidence related to cigarette nicotine content was conducted in 2018, leading up to the meeting convened by the WHO Convention Secretariat. Author ECD was asked to draft a report on the “Behavioral aspects of using cigarettes with reduced addictiveness potential” and was provided an outline of topics to address including: 1) the behavioral implications of reduced addictiveness potential on specific target groups/different sectors of the population taking into consideration the negative behavioral implications and unintended consequences for youth, minors, smokers, non-smokers, other smokers, and social smokers; 2) the potential impact on the market environment including perceptions of new products, manipulation of products by manufacturers or consumers, potential use of other sources of nicotine, and effects on nicotine and non-nicotine replacement therapies; 3) the potential population impact on initiation, relapse, and cessation behaviors. The discussion that ensued at and following the meeting further highlighted the need to focus the review on some common questions/assumptions raised by meeting attendees (paraphrased here): *How might reducing nicotine in cigarettes protect kids who are at risk for or try smoking? Won't people just smoke more and be exposed to more toxicants? Is there evidence that reducing nicotine will help people quit smoking not just reduce how much they smoke? What does the science say about unintended consequences such as nicotine withdrawal, the potential for/extent of an illicit market, exacerbating misperceptions of nicotine as the cause of smoking-related harms, and product manipulation?* and *How might vulnerable populations and other sub-groups be impacted by reduced nicotine cigarettes?* The final review was designed to summarize the available behavioral science addressing these core issues.

Relevant literature was identified in Pubmed and Scopus with the following key search terms: nicotine reduction, very low nicotine content cigarettes, reduced nicotine cigarettes, and nicotine product standards. Only papers written in English were reviewed. 93 papers were identified and reviewed by ECD to determine whether they were within the defined scope of the review as outlined above. The evidence identified was summarized at the meeting in Berlin. That meeting helped shaped a World Health Organization (WHO) technical report entitled “A Global Nicotine Reduction Strategy: State of the Science” (Wayne, Donny & Ribisl, 2019). As more research has been conducted with the goal of informing cigarette nicotine reduction policy, additional literature searches through April 2021 were conducted by CMW using the same search methods as above to identify the additional information included in this review. The search yielded an additional 34 publications that were deemed relevant to this review of behavioral research after screening.

## Results

### Potential benefit: decreased uptake of regular smoking

Reducing the nicotine content of cigarettes could impact the uptake of regular smoking by decreasing the proportion of individuals who first try smoking and by decreasing the probability that individuals who do try smoking progress to regular use. Little is known about how reducing cigarette nicotine content would affect intentions to try cigarettes. Positive expectancies about smoking reinforcement – such as beliefs that smoking will taste good, reduce negative affect, increase energy and attentiveness, and facilitate more enjoyable social interactions – among nicotine-naïve youth are associated with subsequent smoking (Andrews & Duncan, 1998; Copeland & Brandon, 2000). In contrast to positive expectancies, negative expectancies may not be strongly associated with smoking susceptibility, as seen in one study with adolescents (Dalton, Sargent, Beach, Bernhardt, & Stevens, 1999). While it seems likely that reducing nicotine in cigarettes will reduce the extent to which people expect a positive smoking experience, to our knowledge, no studies have assessed how nicotine reduction impacts expectancies among people naïve to cigarettes and the effects of nicotine. It is also unclear how positive smoking expectancies are attributed to nicotine vs. non-nicotine effects. Among those who do go on to try very low nicotine cigarettes, however, the likelihood that experimentation transitions to regular use depends on the reinforcing effects (or lack thereof) actually experienced. The ability to reliably discriminate a dose of drug from a placebo, the subjective effects of use, and behavioral measures of motivation to use are all indicative of a substance's reinforcing efficacy. Evidence gathered across these measures suggest very low nicotine cigarettes are less reinforcing than regular cigarettes, as described next.

Discriminating, or being able to feel and notice the effects of nicotine may be necessary for drug reinforcement and promote chronic use. When asked to choose between a 0.4 mg/g nicotine cigarette (a “very low nicotine cigarette”) and cigarettes with various nicotine contents ranging from 1 to 17 mg/g nicotine, participants who sampled each product but were blind to the nicotine content favored cigarettes with greater nicotine content when they could detect a difference (Perkins et al., 2017). Discrimination of nicotine in cigarettes is dose-dependent; the median nicotine content distinguishable from 0.4 mg/g is 11 mg/g, although there is a wide degree of heterogeneity with some individuals able to reliably detect nicotine contents as low as 2.4 mg/g (vs. 0.4 mg/g) (Perkins, Kunkle, Karelitz, Michael, & Donny, 2016). Discrimination effects are similar for individuals both nicotine dependent and not nicotine dependent, suggesting these effects may generalize to people who have not yet transitioned to regular smoking (Perkins et al., 2017). Together, these data suggest that reducing nicotine below 2.4 mg/g could reduce the transition to regular use in the greatest number of people who experiment with smoking.

The subjective effects of early smoking experiences are associated with an increased likelihood of subsequent use (Pomerleau, Pomerleau, & Namenek, 1998). Effects such as getting a “buzz”, or a boost of pleasure and energy, are attributed to nicotine delivery and associated with an increased risk of regular smoking. Reduced nicotine content could therefore blunt initial, pleasurable experiences (Perkins et al., 2009). Following brief sampling of low nicotine cigarettes or extended periods of use, studies find that participants who smoke regularly rate low nicotine cigarettes as less satisfying, less rewarding, less enjoyable, less able to reduce craving (Cassidy et al., 2019; Smith, Donny et al., 2019). This attenuation of positive subjective effects may be greatest in policy scenarios that reduce nicotine content to very low levels right away, rather than policy scenarios that gradually step down nicotine content over time (Lin et al., 2020; Smith, Donny et al., 2019). Lastly, simply telling people who smoke that nicotine is reduced can significantly lower the subjective effects people report when smoking after abstinence, regardless of actual nicotine content (Juliano, Fucito, & Harrell, 2011). Hence, early smoking experiences are likely shaped by both the actual and perceived nicotine content of cigarettes.

Behavioral indices of the motivation to use cigarettes with varying levels of nicotine provide additional evidence as to whether reducing nicotine content is likely to disrupt the transition from experimentation to regular use. Using a classic paradigm for evaluating a substance's acute reinforcing value, researchers have shown that low nicotine cigarettes maintain self-administration during a brief period similar to conventional cigarettes when they are the only option available (Shahan, Bickel, Madden, & Badger, 1999; Shahan, Bickel, Badger, & Giordano, 2001). Similarly, if smoking is contingent on completing a laboratory task that gets progressively harder with each puff earned, participants just introduced to lower nicotine cigarettes earn a similar number of puffs as individuals assigned to normal cigarettes. However, with repeated use lower nicotine cigarettes result in fewer puffs earned indicating reduced motivation to smoke (Donny, Houtsmuller, & Stitzer, 2007). These latter data are consistent with data derived from behavioral economic methods, which typically assess hypothetical purchasing of cigarettes across a wide range of prices, capture multiple aspects of an individual's demand for cigarettes (independent of physical effort or sustained attention) and are strongly associated with actual cigarette consumption (Cassidy & Kurti, 2018; González-Roz, Jackson, Murphy, Rohsenow, & MacKillop, 2019). Studies show that cigarettes with lower nicotine content produce less economic demand (Davis et al., 2019; Higgins et al., 2017). Relative to normal nicotine cigarettes, consumption of low nicotine cigarettes is lower and more participants from extended access clinical trials anticipate abstaining across a wider range of prices (Smith, Rupprecht et al., 2017). Together, these data suggest that low nicotine cigarettes are less likely to sustain regular use compared to normal nicotine cigarettes.

A limitation of the clinical studies assessing the reinforcing nature of low nicotine cigarettes is that, for clear ethical reasons, they have been conducted in people who smoke regularly; therefore, the impact of reducing nicotine in people relatively naïve to cigarettes and naïve to nicotine is unknown. However, animal research using self-administration - the most widely accepted method for testing drug reinforcement - have demonstrated that lower doses decrease the proportion of rodents that reliably self-administer nicotine and increase the amount of time it takes to establish self-administration (Schassburger et al., 2016; Shram, Li, & Lê, 2008; Smith, Schassburger, Buffalari, Sved, & Donny, 2014). These effects have been observed in adolescent and adult nicotine naïve animals and support the notion that reducing nicotine in cigarettes would limit the uptake of regular smoking behavior among people trying low nicotine cigarettes.

Arguably, a more useful test of the potential real world impact on behavior is to examine the value of a commodity relative to possible alternative behaviors. When presented with choices between concurrently available low and normal nicotine content cigarettes, participants demonstrate strong preferences for normal nicotine content, suggesting lower nicotine content reduces relative value and that demand for regular cigarettes could remain strong (Perkins, Jacobs, Sanders, & Caggiula, 2002; Perkins et al., 2016; Shahan et al., 1999). A critical question for policy analysts that may prove difficult for behavioral scientists to address is whether the availability of non-combusted alternatives to low nicotine cigarettes will significantly reduce demand for regular cigarettes that would only be available from an illicit market, including amongst youth.

A primary goal of reducing nicotine in cigarettes is to prevent chronic smoking. Reducing nicotine may make individuals less likely to try smoking if it reduces positive expectancies. It may also lessen the likelihood of transitioning to regular use because it makes cigarettes inherently less reinforcing, although the behavioral impact may depend on what other products are available to users. These predictions are based primarily on adults who smoke regularly and experimental animals.

### Potential benefit: decreased smoking intensity

Reducing nicotine in cigarettes would benefit public health if it effectively reduces how much people smoke. This potential benefit contrasts one of the most commonly voiced concerns about reducing nicotine, the potential for compensatory smoking. Concerns about compensation (i.e., increases in smoking intensity to “offset” reductions in nicotine) are based, in part, on the history of light cigarettes. Light cigarettes, which are ventilated to reduce machine-generated nicotine yields, but contain normal amounts of nicotine in the tobacco, are known to produce compensation (Benowitz et al., 2005; Bernert et al., 2005; Hecht et al., 2005; Mendes, Kapur, Wang, Feng, & Roethig, 2008). People who smoke regularly can and do adjust their behavior (e.g., smoke more cigarettes, take larger puffs, block ventilation holes) to effectively maintain nicotine exposure while smoking light cigarettes.

Multiple randomized control trials have investigated how nicotine reduction affects the number of cigarettes smoked and smoke exposure. For example, Donny et al. (2015) randomly assigned adults to smoke their usual brand cigarettes or experimental cigarettes across a range of doses for six weeks. Those assigned to the lowest doses smoked approximately 25–40 percent fewer cigarettes per day at the end of the study compared to those assigned their usual brand or the normal nicotine-content investigational cigarettes. In another trial, Hatsukami et al. (2018) compared the effects of immediate and gradual nicotine reduction against a control group. Those randomly assigned to the immediate reduction group similarly smoked fewer cigarettes per day and had corresponding declines in biomarkers of nicotine and smoke exposure. It is important to note that if nicotine is only moderately reduced (e.g., 5.2 mg/g), compensatory smoking may be more likely to occur (Hatsukami et al., 2018; Hatsukami, Perkins et al., 2010). This evidence suggests that smoking intensity may be nicotine-dose dependent, and decreases following substantial reductions in nicotine content. The extent to which cigarettes per day declines with very low nicotine cigarette use in these trials may represent a conservative estimate of the cigarette per day reductions that would occur following the implementation of a nicotine reduction policy, as participants in these studies receive cigarettes at no cost and, at least in initial trials, were asked not use alternative nicotine sources that would be available to them in the real world.

A limitation of the large trials simulating a nicotine reduction policy scenario is that participants ultimately still have easy access to regular-nicotine content, non-investigational cigarettes. Non-adherence is common and could mask compensatory behavior that would otherwise occur. However, other findings suggest compensation is unlikely even when regular nicotine cigarettes are unavailable. Brief laboratory studies of smoking behavior suggest that although increases in puffing behavior may occur for the first few low nicotine cigarettes smoked, those attempts at compensation quickly dissipate (MacQueen et al., 2012). Furthermore, studies conducted in controlled, inpatient settings, where exclusive low nicotine cigarette use is enforced, also fail to find signs of compensation. For example, one study found that smoking declined by approximately five cigarettes per day with an accompanying decrease in carbon monoxide when non-treatment seeking participants used low nicotine cigarettes while residing on an inpatient unit for 11 days (Donny et al., 2007). In another recent residential study, Smith et al. (2020) aimed to more accurately capture the effects of a real-world nicotine reduction scenario. The authors provided participants with exclusive access to investigational cigarettes at a cost and open-label, in contrast to trials where cigarettes are often provided for free and nicotine content is blinded. Comparing within-participant data from four days with exclusive access to low nicotine content cigarettes and four days with exclusive access to normal nicotine content cigarettes, they did not observe increases in cigarette per day nor lasting increases in biomarkers of smoke exposure. Maintaining usual nicotine intake by compensating with 0.4 mg/g cigarettes would be difficult, even with drastic changes in smoking intensity that would likely be aversive. Future behavioral research should determine how nicotine reduction in cigarettes affects smoking intensity within the context of a realistic marketplace.

### Potential benefit: decreased dependence and increased cessation

Reducing the nicotine content of cigarettes could also increase the occurrence and success rate of quit attempts. The majority of clinical trials investigating cigarette nicotine reduction have been conducted with participants who are *not* currently interested in quitting or seeking cessation services. Benowitz and colleagues found that individuals assigned a low nicotine cigarette, after six months of progressive nicotine content reduction, were more intent on quitting (less likely to be in the “pre-contemplation stage”), although only a few participants actually made a quit attempt (Benowitz et al., 2012). In the Donny et al. (2015) trial previously described, participants were assigned to use cigarettes with one of six different nicotine doses for six weeks. Those assigned the lowest nicotine content (0.4 mg/g) were significantly more likely to have made a quit attempt within 30 days of completing the study, compared with those randomized to the highest nicotine yield cigarettes. In a follow-up study, Smith, Donny et al. (2019), found use of very low nicotine cigarettes, with or without a patch, did not increase abstinence during a “practice quit attempt;” however, secondary, causal inference, analyses that estimated abstinence rates if all participants had been adherent indicated that exclusive use of low nicotine cigarettes increases the ability to abstain from smoking. In a relatively small study, Walker et al. (2015) either provided participants with a free 12-week supply of low nicotine cigarettes or allowed participants to purchase their usual brand cigarettes. Those provided cigarettes with reduced nicotine content were more likely to make a quit attempt across the 12-weeks. Data from the largest clinical trial to date by Hatsukami et al. (2018) found that participants randomized to receive 0.4 mg/g nicotine cigarettes for 20 weeks had more smoke-free days during the trial and were more likely to be abstinent from smoking (7-day; confirmed with breath carbon monoxide) at the end of the study. Finally, a trial compared changes in dependence between those who gradually transitioned to low nicotine cigarettes with those instructed to make equivalent reductions in cigarettes per day (Klemperer, Hughes, Callas, Benner, & Morley, 2019). Interestingly, the low nicotine cigarette condition reduced average dependence after 4 weeks to a greater extent than cutting out cigarettes. This suggests smoking low nicotine cigarettes may uniquely affect dependence by uncoupling smoking related cues from pharmacological reward, disrupting conditioning.

Other trials have investigated the effects of cigarette nicotine reduction in participants actively trying to quit smoking. Rezaishiraz, Hyland, Mahoney, O'Connor and Cummings (2007), assessed quitting in participants who smoked 20+ cigarettes per day and found that 2 weeks of use of low nicotine cigarettes combined with transdermal nicotine, compared to transdermal nicotine alone, reduced cravings but did not increase quit rates. Becker, Rose and Albino (2008), found that use of low nicotine cigarettes and a nicotine patch was more effective in helping participants achieve 4 weeks of continuous abstinence than use of normal nicotine cigarettes and the nicotine patch. Interestingly, participants reporting complete adherence to the use of the reduced nicotine cigarettes reported greater abstinence. Similarly, Hatsukami et al. found that individuals who smoked light cigarettes and were interested in quitting were more likely to be abstinent during a 6-week follow-up period if they had been randomized to use the lowest nicotine content cigarette relative to a cigarette with intermediate levels of nicotine (Hatsukami, Perkins et al., 2010); in a subsequent study, very low nicotine content cigarettes produced abstinence rates comparable to nicotine replacement (Hatsukami et al., 2013). Similar to Becker, a secondary analysis of the biomarker data from the two Hatsukami trials found that individuals with the greater reductions in nicotine exposure prior to quitting were more likely to be abstinent at follow-up (Dermody, Donny, Hertsgaard, & Hatsukami, 2015), again suggesting that adherence with the use of low nicotine cigarettes is associated with increased likelihood of achieving abstinence.

Assessing the potential impact of a product standard on cessation outcomes is difficult within the context of clinical trials. Often, participants do not intend to quit when enrolled, they receive low nicotine cigarettes for a limited amount of time (with knowledge that they will be able to return to their usual brand at study completion), and they have access to widely available regular cigarettes that limits adherence. Many studies are also likely underpowered statistically; intervention conditions typically have fewer than 100 people (Hatsukami et al., 2018 is the exception). Therefore, in addition to considering quitting behaviors within the context of clinical trials, we also provide an overview of what is known about the impact of low nicotine cigarettes on indices of cigarette dependence as a potentially important indicator of smoking cessation.

Two widely used dependence assessments shown to correlate with withdrawal and cessation likelihood include the Brief Wisconsin Inventory of Smoking Dependence Motives (WISDM) and Fagerström Test for Cigarette Dependence, FTCD (Fagerstrom, 2012; Piper et al., 2008; Smith et al., 2010). Multiple clinical trials have found that after at least six weeks, participants assigned to cigarettes with 0.4 mg/g nicotine had lower WISDM and FTCD scores than those assigned to normal nicotine-content cigarettes, suggesting decreased dependence (Donny et al., 2015; Hatsukami et al., 2018). On average, participants who use low nicotine cigarettes over an extended period of time also reported greater average reductions in craving and withdrawal symptoms following brief abstinence compared to participants using normal nicotine content cigarettes (Donny et al., 2015).

Overall, indices of reinforcing efficacy, cigarette dependence, quit attempts, and abstinence indicate that large reductions in the nicotine content of cigarettes could increase smoking cessation. This may be most likely when the nicotine content is reduced below 2.4 mg/g. In Donny et al. (2015), cigarettes with 0.4 mg/g produced the clearest decreases in dependence and increase in quit attempts. These findings are consistent with other studies indicating that some people who smoke can discriminate between cigarettes with 2.4 and 0.4 mg/g (Perkins, 2019; Perkins et al., 2016). Therefore, setting limits at or below 0.4 mg/g or less would likely maximize the effect of nicotine reduction on cessation. Finally, it is important to note that when participants in clinical trials have been asked what they would do if low nicotine cigarettes were the only cigarettes available to them, over 50% of participants assigned to the 0.4 mg/g condition indicated that they would stop smoking within 1 year (Smith, Cassidy et al., 2017) compared to less than 20% of participants assigned cigarettes with 15.8 mg/g nicotine. Qualitative interviews with participants in a residential study who used open-label low nicotine cigarettes exclusively for 4 days generated a variety of responses to questions about predicted behavior in a real nicotine reduction policy scenario; with some people stated they would taper off and quit, and other stating they would switch to other nicotine sources or seek out black market normal nicotine cigarettes (Denlinger-Apte et al., 2021).

### Potential harm: withdrawal, the use of other combusted nicotine products, and the black market

Substantially reducing nicotine content in cigarettes may result in withdrawal symptoms among people who smoke. After 5 days of use, Buchhalter, Acosta, Evans, Breland and Eissenberg (2005) found that low nicotine cigarettes effectively suppressed some symptoms of withdrawal, most notably urges to smoke, but not other symptoms including those related to attention, restlessness, and increased eating. Secondary analyses of the Donny et al. (2015) showed that relative to participants assigned normal nicotine cigarettes, those using very low nicotine cigarettes were more likely to experience increased anger, irritability, frustration and increased appetite during the initial weeks of the study, but the significant elevation of all symptoms was resolved by the end of the six-week trial, no longer differing between groups (Dermody et al., 2018). Reductions in nicotine exposure may lead to weight gain in some individuals, similar to the levels observed following smoking cessation (Rupprecht et al., 2017)

The severity of withdrawal and the impact on behavior will likely depend on the extent to which nicotine exposure is reduced. In the(Hatsukami et al., 2018) comparing gradual and immediate nicotine reduction implementation strategies, the immediate group had lower cigarette consumption and toxicant exposure across time, greater reductions in dependence, and more cigarette-free days, but also experienced greater withdrawal symptoms. In these trials, those assigned to switching abruptly to low nicotine cigarettes show greater use of non-study cigarettes (non-adherence) and higher drop-out rates, which may reflect both an attempt to reduce withdrawal symptoms and the lack of satisfaction with low nicotine cigarettes. If a mandated reduction in nicotine content were implemented and regular nicotine content cigarettes were not available, people who smoke may be motivated to hoard normal nicotine cigarettes, tamper with low nicotine products (e.g. trying to add nicotine to their cigarettes by dripping e-liquids on tobacco), and/or seek out normal nicotine cigarettes in an illicit market. Though hoarding would only delay implementation and therefore is a minor concern with few long-term consequences, product tampering and illicit purchasing could create new risks. To our knowledge, no studies have evaluated the likelihood of tampering or the potential changes in toxicity compared to current cigarettes. Prohibition can generate a black market for a drug or substance that greatly reduces the anticipated reductions in use. In a cigarette nicotine reduction policy cigarettes are not prohibited, and neither is nicotine delivery, though some still consider it a functional cigarette prohibition (Kozlowski, 2017). Some people who smoke, particularly those with high levels of cigarette dependence, indicate willingness to seek out illicit cigarettes in response to a nicotine reduction policy (Denlinger-Apte et al., 2021; Hall, Byron, Brewer, Noar, & Ribisl, 2019; Patel et al., 2019). Ribisl and colleagues reviewed strategies to reduce the potential demand for illicit cigarettes that could emerge in response to a reduction in the nicotine content of legal cigarettes (Ribisl, Hatsukami, Huang, Williams, & Donny, 2019). A more thorough analysis of the similarities and differences between a nicotine reduction policy and other interventions that have impacted illicit markets is warranted.

In addition to product tampering and illicit market demand, people who smoke may cope with nicotine reduction in cigarettes by seeking out nicotine in other sources. Nicotine replacement therapy (NRT), for instance, may reduce the withdrawal associated with nicotine reduction (Hatsukami et al., 2013). Accordingly, concurrent use of transdermal nicotine with reduced nicotine cigarettes could facilitate the decrease in smoking as described above (Donny & Jones, 2009, Hatsukami et al., 2010, Smith et al., 2019). Countries considering nicotine reduction should have adequate infrastructure in place to meet potential increases in demand for NRT and other non-combusted alternatives like e-cigarettes.

Relatively little is known about the effects of cigarette nicotine reduction in the context of nicotine alternatives other than NRT. An important concept for anticipating the use of other products is relative reinforcement. Reducing nicotine in cigarettes will likely increase the relative appeal of some non-medicinal nicotine-containing products. In a pilot study by Hatsukami and colleagues (2017), participants could purchase nicotine products from a marketplace with either low nicotine cigarettes or normal nicotine cigarettes. Those assigned to the low nicotine cigarette condition used more non-cigarette products, and products that resemble cigarettes in nicotine delivery and sensorimotor characteristics were the most likely alternatives to be used.

Substitutability and toxicity should be carefully considered when thinking which products should be subject to mandated nicotine reduction. Combusted products similar to cigarettes in both nicotine and toxicant delivery and known to function as substitutes for cigarettes (e.g. little cigars and cigarillos) should be included in standards restricting nicotine content. Failure to include these products would likely lead to switching from cigarettes to another combusted tobacco product with similar health consequences (Byron, Strasser, & Delnevo, 2019, Delnevo, Giovenco, & Miller Lo, 2017, Delnevo, Hrywna, Giovenco, Miller Lo, & O’Connor, 2017, Gammon et al., 2016, Zheng, Zhen, Dench, & Nonnemaker, 2017). In contrast, maintaining access to cigarette-like nicotine reinforcement in non-combusted products, such as e-cigarettes, could potentially mitigate withdrawal symptoms and provide a satisfying alternative, while effectively reducing harm to people who smoke. Consequently, the public health impact of reducing nicotine in cigarettes will likely depend on what alternative nicotine products are available to people who smoke (Hatsukami et al., 2017; Abrams & Notely, 2020; White, Hatsukami, & Donny, 2020).

### Potential harm: misperceptions of harm and the need for effective health communications

Low nicotine cigarettes remain extremely harmful if smoked similarly to regular cigarettes; however, misperceptions about the direct harms caused by nicotine could lead people to believe they are less harmful. For instance, many incorrectly believe nicotine is the primary cancer-causing agent in cigarettes (O'Brien, Nguyen, Persoskie, & Hoffman, 2017). In a national sample of people who smoke in the U.S., more than 47% believed using low nicotine cigarettes for 30 years would be less likely to cause cancer than smoking their usual cigarettes (Byron, Hall, King, Ribisl, & Brewer, 2019). Almost one quarter reported they would be less likely to quit if only low nicotine cigarettes were available, presumably because they would be less concerned about the health risks. Furthermore, individuals who believe or are told they are using low nicotine cigarettes report lower harm perceptions than those who believe or are told they are using normal nicotine cigarettes, regardless of actual cigarette nicotine content (Denlinger-Apte, Joel, Strasser, & Donny, 2016; Mercincavage, Smyth, Strasser, & Branstetter, 2016). Exposure to ads from previously marketed low nicotine cigarettes can increase false assumptions about reduced harm (Shadel et al., 2006; Strasser, Tang, Tuller, & Cappella, 2008). In one study among a sample of U.S. adults who smoke, participants were asked to provide their perceptions of low nicotine cigarette harm and addictiveness (Mercincavage et al., 2019) In this sample, the majority of participants (correctly) disagreed with statements about low nicotine cigarettes being safer than regular cigarettes. Most participants also (incorrectly) disagreed with statements that low nicotine cigarettes are less addictive and could making quitting smoking easier. A substantial proportion of participants also indicated they were unsure whether they agreed or disagreed with these statements.

The behavioral consequences of perceptions of risk and/or addictiveness are not clear. Theoretically, misperceptions of reduced risk could contribute to initiation among people who have not used cigarettes, and relapse among people who formerly smoked. For people who smoke, misperceptions may limit reductions in smoking intensity and/or limit the increased the likelihood of cessation, but misperceptions are unlikely to completely undermine these effects. Indeed, even participants who reported reduced risk perceptions after six weeks of low nicotine cigarette use used fewer cigarettes and reported being less likely to persist in using the product one year later relative to the control condition (Pacek et al., 2018).

Uncertainty and misunderstandings about the health risk and addictiveness of low nicotine cigarette use suggest educational campaigns have an important role to play in helping consumers evaluate these products and any nicotine reduction policy. A study by Villanti et al. (2019) found that a brief nicotine messaging intervention (viewing statements like: “*nicotine is the addictive substance in tobacco products*,” “*nicotine makes it harder for people to quit smoking*,” and “*nicotine does not cause cancer*”) effectively correct nicotine misperceptions in an online sample. Byron and colleagues tested how specific descriptors of reduced nicotine content affected harm and addictiveness perceptions about low nicotine cigarettes (Byron, Hall, King, Ribisl, & Brewer, 2019). Message conditions that included a percentage (e.g. “95% of nicotine would be removed”) resulted in the most accurate perceptions about low nicotine cigarettes’ nicotine content and addictiveness, but less accurate perceptions about cancer risk. Achieving accurate perceptions may require messages that both communicate about the intent of reducing nicotine (to reduce addictiveness) and the harm caused by smoking regardless of nicotine content (harm misperceptions) (Popova et al., 2019). A recent focus group study found that people who smoke are receptive to messages about low nicotine cigarettes increasing quitting efficacy and messages about low nicotine cigarettes being equally as harmful traditional cigarettes (Duong et al., 2021). Among participants who used to smoke or who have never smoked, however, there were concerns about the messages downplaying smoking risks and potentially inspiring initiation and relapse. Another recent study randomized an online, crowdsourced sample of people who smoke to view low nicotine cigarette advertisements with different features. Ads with the disclaimer “*Nicotine is addictive. Less nicotine does NOT mean a safer cigarette. All cigarettes cause disease and death*,” resulted in greater risk perceptions and fewer false believes than identical ads with no disclaimer (Mercincavage et al., 2021). The same study also found that when the advertised low nicotine cigarettes had the brand name “*Moonlight*” (containing the misleading descriptor “light”) participants perceived lower health risks than those that saw the brand name “*Moonrise*”. Together, this evidence suggests public health messaging and industry advertising have the potential to minimize or exacerbate confusion about low nicotine cigarette risk.

Misperceptions about the harms caused by nicotine are pervasive. As a result, people may assume that cigarettes with reduced nicotine are safer to use. These misperceptions may be exacerbated by product advertising if not restricted. Regulating nicotine content has the potential to worsen misperceptions given the role that government plays in ensuring the safety of products. Therefore, advertising, promotion and sponsorship should be restricted as much as possible within the limits imposed by governmental bodies, and health communication campaigns should be designed to make clear that nicotine is responsible for continued use and addiction, and that combustion is responsible for the deadly harms of tobacco use. This means ensuring the public understands that the persistent use of low nicotine cigarettes still poses a grave risk to health; and that switching to some alternative, non-combusted, legal sources of nicotine may reduce risk.

### Potential harm: manipulation of products and the need for additional regulation and/or surveillance

Manufacturers could attempt to alter the content or design of low nicotine cigarettes to render them more satisfying and possibly more addictive. Changes in cigarettes that attempt to increase the transfer of nicotine from the product to the person (e.g., pH) would likely be ineffective given that the tobacco filler would contain very little nicotine and additional gains in yield are unlikely to offset a large decrease in content. Nevertheless, standards could be extended to the nicotine yield of the product (e.g., <0.05 mg). Other constituents of tobacco (e.g., other alkaloids, beta-carbolines, acetaldehyde) have the potential to impact smoking behavior, although preclinical data suggest that these compounds would have to be present in much higher levels than those currently observed in cigarettes to support behavior in the absence of reinforcing doses of nicotine (Smith, Rupprecht et al., 2017; White, Pickworth, Sved, & Donny, 2019). Compounds that inhibit monoamine oxidase, an enzyme that metabolizes monoamines like dopamine and serotonin, can increase reinforcement from low doses of nicotine; however, if nicotine is reduced enough, self-administration still declines (Smith et al., 2015; Smith et al., 2016). Finally, flavors, which have their own intrinsic appeal, could perpetuate use in the absence of nicotine. For example, menthol can mask initially aversive aspects of smoking and become a reinforcing sensory cue over time (Wickham, 2020). We are not aware of data testing the effects of other flavorants in cigarettes or other combusted tobacco products.

Regulators should take into account the possibility that nicotine exposure/effect could vary by both product characteristics (e.g., pH) and individual variability (e.g., nicotine metabolism), setting nicotine levels as low as possible. It may also be prudent to establish a complementary standard for nicotine yield (established under a variety of testing conditions) to minimize risk that manufacturers would attempt to maintain nicotine exposure despite the reduced nicotine content in the tobacco itself. Hence, effective implementation of a low nicotine product standard would require capacity to monitor nicotine levels to ensure compliance by manufacturers. Product standards for other constituents and/or design features might be prudent; however, there are no clear examples of product features that could maintain or boost cigarette reinforcement if nicotine is adequately reduced.

### The impact of nicotine reduction in notable sub-populations of people who smoke

The potential benefits of reducing nicotine in cigarettes are often discussed in the context of net-population impact. However, it is important to anticipate potential heterogeneity in responses to nicotine reduction, to look at the effects of cigarette nicotine content in various priority sub-groups of people who smoke. While a growing literature addresses a number of different populations of interest, additional research among some sub-groups, such as people living with HIV (Denlinger-Apte et al., 2019) and people who are pregnant (Heil et al., 2020) would further address this concern.

#### People with psychiatric comorbidities

People with serious mental illness smoke at higher rates than the general population and experience particularly high levels of craving and negative affect during abstinence. A “self-medication” hypotheses postulates that people with psychiatric comorbidities find nicotine particularly reinforcing because it can modulate mood and mitigate other disruptive symptoms. Accordingly, several studies have investigated whether people who smoke with depression, schizophrenia, schizoaffective disorder, or bipolar disorder respond differently to nicotine reduction in cigarettes than people without serious mental illness (Higgins et al., 2017; Higgins et al., 2020; Tidey et al., 2017; Tidey et al., 2019). The available empirical literature provides support for the idea that the potential benefits of a reduced-nicotine standard for cigarettes that have been observed among people without psychiatric disorders would extend to people with mental health conditions such as depression, with these individuals demonstrating reduction in cigarette use without signs of compensation (Denlinger-Apte et al., 2020, Tidey et al., 2017). In a literature review, Gaalema et al. (2019) concluded that although disruption of mood following withdrawal from nicotine may be greater in people with affective disorders, “use of very low nicotine content cigarettes during abstinence may help mitigate the mood-disrupting effects of initial abstinence” and longer-term abstinence may produce “psychiatric improvement rather than worsening.” It is worth noting that in one study by Tidey et al. (2019), a sample of people with serious mental illness assigned to low nicotine cigarettes decreased cigarette consumption and had lower carbon monoxide levels than those assigned to normal nicotine cigarettes; however, biomarkers of nicotine exposure did not differ between the two groups. This suggests participants in the low nicotine cigarette condition obtained nicotine from non-combusted sources not provided by the study. Thus, people in this population may be expected to seek out other forms of nicotine if nicotine in cigarettes is reduced. Heterogeneity in the extent to which the results observed in existing studies would extend to people living with mental illness deserves further attention.

#### People with low socioeconomic status (SES)

The burden of tobacco related disease is disproportionately distributed among economically disadvantaged populations, such as people who are unemployed, unhoused and less educated. On average, this population also experiences higher nicotine dependence and lower motivation to quit. A few trials have intentionally examined how people with low SES who smoke respond to nicotine reduction in cigarettes. Higgins et al. (2017), Higgins et al. (2020) reported that when participants with low socioeconomic status switch to cigarettes with 2.4 mg nicotine per gram of tobacco or 0.4 mg nicotine per gram, their smoking rates and nicotine-dependence severity drops. Krebs and colleagues found that when undergoing nicotine reduction in a step-down approach over 33 weeks, low SES individuals smoked less and had lower biomarkers of smoke exposure as intended (Krebs et al., 2021). However, those assigned to low nicotine cigarettes were less likely than those assigned to usual nicotine-content cigarettes to adhere to only using the cigarettes assigned by the study, and only those who were adherent made quit attempts between the end of the study and a follow-up visit (Higgins et al., 2020). These results emphasize a need for affordable and accessible alternative, non-combusted nicotine sources to minimize unintended consequences among this population.

#### People who use other substances

People who use other drugs may not experience the same benefits of nicotine reduction and/or may increase their use of other substances in response to nicotine reduction. However, secondary analyses of nicotine reduction clinical trials find that immediate reduction to very low nicotine content reduces smoking intensity without producing increases in alcohol consumption, including among people who drink heavily and among people who drink that are especially dependent on nicotine (Dermody et al., 2016; Dermody et al., 2020). In fact, among some participants assigned to low nicotine cigarettes for 20 weeks, binge drinking decreased, suggestive of a potential decoupling of the daily associations between smoking and drinking (Dermody et al., 2020). Similar analyses on marijuana use suggest that switching to low nicotine cigarettes is likely to benefit people regardless of marijuana use status and is unlikely to alter the prevalence or frequency of cannabis use (Pacek et al., 2016). Finally, nicotine reduction clinical trials enrolling people who use opioids found that this smoking population also experienced lower smoking rates and decreased nicotine dependence severity after using low nicotine cigarettes, relative to normal nicotine cigarettes (Higgins et al., 2017, Higgins et al., 2020; Streck et al., 2020). Overall, evidence does not support that nicotine reduction affects people who use other drugs uniquely, nor that nicotine reduction leads to the compensatory use of other substances.

#### Youth and young adults

Some preclinical data suggests that adolescence may be a period of heightened sensitivity to nicotine reinforcement (Levin et al., 2007). A few studies have assessed the impact of nicotine reduction on young adults who smoke. Kassel et al. (2007) showed that regular cigarettes suppress negative affect in adolescents who smoke, but that this effect was attenuated when they used a low nicotine cigarette. Faulkner et al. (2017) found that young adults who smoke reported fewer positive effects of low nicotine cigarettes, and experienced temporary attentional deficits compared to higher nicotine cigarettes. Cassidy et al. (2018) conducted a secondary analysis of young adults who used low nicotine cigarettes for six weeks in Donny et al. (2015) and found that low nicotine cigarettes were associated with significantly lower positive subjective effects and fewer cigarettes per day than older adults after two weeks of use, indicating that regulations that reduce nicotine content in cigarettes may reduce smoking reinforcement relatively quickly in young adults. A similar secondary analysis of Hatsukami et al. (2017) found that cigarettes per day was significantly lower in the group assigned to an immediate reduction to very low nicotine cigarettes (vs. gradual nicotine content reduction and the control condition) for those between 18 and 25 years old and those older than 25 (Cassidy et al., 2021). As the vast majority of people who have smoked for years initiated during adolescence and young adulthood (Centers for Disease Control and Prevention (US) 2010), policies that minimize the addictiveness of cigarettes in these populations could have a marked impact on smoking prevalence.

#### People who smoke infrequently

A substantial percentage of people use cigarettes less than daily and/or smoke only a few cigarettes a day. This smoking pattern contrasts with those most often recruited for nicotine reduction research – people who smoke 5+ cigarettes daily. Although their smoking is not confined to social situations, these individuals are often called “social smokers,” and they differ in a number of ways (e.g., dependence symptoms) that could contribute to differences in response to reduced nicotine cigarettes (Shiffman, 2009). Nevertheless, trials focused on people who use cigarettes non-daily found a significant (>50%) decrease in cigarettes per day as a consequence of cigarette nicotine reduction, suggesting they may experience similar benefits compared to people who smoke more heavily (Shiffman, Kurland, Scholl, & Mao, 2018; Shiffman, Mao, Kurland, & Scholl, 2018; Shiffman, Scholl and Mao, 2019).

#### People who smoke menthol cigarettes

Investigations of the flavorant menthol suggest that menthol can influence the appeal and reinforcing value of tobacco products through multiple mechanisms. For example, menthol can mask initially aversive aspects of smoking and become reinforcing sensory cues over time (Villanti et al., 2016; Wickham et al., 2020). Animal studies further suggest that menthol may interact with nicotine to directly affect reward pathways in the central nervous system (Cooper & Henderson, 2020; Jao et al., 2020). In the United States there are also cultural factors associated with using menthol cigarettes. For example, African American race and menthol preference are highly overlapping (Trinidad, Perez-Stable, Messer, White, & Pierce, 2010). These factors raise questions about whether the effects of nicotine reduction could differ between people who smoke menthol and non-menthol products. A secondary analysis of the Hatsukami et al. (2018) trial found that menthol preference moderated the effect of immediate nicotine reduction on cigarettes per day and expired carbon monoxide, such that people who smoke menthol cigarettes reduced their smoking but to a lesser extent than people who smoke non-menthol cigarettes (Denlinger-Apte et al., 2019). Another recent study found no differences in responses to nicotine reduction by menthol preference across measures of economic demand, withdrawal or smoking topography (Davis et al., 2019). Though findings are somewhat mixed as to whether and how menthol moderates the extent of nicotine reduction's effects, both people who smoke menthol and non-menthol cigarettes appear to benefit in terms of reductions in smoking intensity.

## Conclusions

For decades, nicotine has been recognized as the primary source of reward and addiction in toxic combusted tobacco products. A growing body of behavioral science suggests that reducing the level of nicotine in cigarettes could render them less reinforcing. Nicotine reduction thus holds the promise of potentially decreasing the probability that naïve young people start using cigarettes regularly, and potentially increasing the probability that people who currently smoke will quit. However, even given the largely consistent behavioral findings, simple straight lines cannot necessarily be drawn between a study using low nicotine cigarettes and real world policy scenario outcomes.

After enactment of a nicotine reduction policy, whether someone who smokes responds by continuing to use low nicotine cigarettes, by seeking out illicit regular nicotine cigarettes, or by quitting smoking could be heterogeneous, depending on a wide variety of combinations of personal and contextual factors not accounted for existing behavioral studies. Particularly important are the accessibility and endorsement of alternative nicotine products and cessation support. Extending the product standard to other combusted tobacco products that effectively substitute for cigarettes (i.e. little cigars), maintaining access to non-combusted forms of rapid nicotine delivery, and educating people who smoke about their options could possibly maximize the potential benefits of a cigarette nicotine reduction policy and decrease black market demand (Abrams & Notley, 2020; Smith, Koopmeiners et al., 2019; Benowitz, Donny, & Hatsukami, 2016; Byron, Strasser, & Delnevo, 2019). Rather than a prohibition on nicotine or on cigarettes, nicotine reduction in combusted products should establish a clear goal to reduce harm by favoring cleaner sources of nicotine delivery. Another important consideration is that laudable policy efforts aiming to improve a given health outcome at the net-population level can create or exacerbate harmful health disparities along the way (Frohlich & Potvin, 2008). Without careful consideration for factors such as the broader regulatory landscape, harm perceptions, and the prioritization of reducing health disparities, a nicotine reduction product standard could foster this kind of heterogeneous outcome. For example, hypothetically, nicotine reduction in cigarettes could drive historically oppressed sub-populations of people who smoke to the black market where they are subject to the harms of product contamination and discriminatory, violent prosecution, while other people who smoke achieve cessation. We would strongly suggest that implementation of a nicotine reduction policy target manufacturing, distribution, and commercial sales and not criminalization of individuals for possession of illicit cigarettes. Given that net-population impact and the reduction of health disparities can be at odds with each other, how nicotine reduction serves these goals should be examined.

In conclusion, behavioral evidence suggests that reducing cigarette nicotine content can render these harmful products less reinforcing. Product standards capping the amount of nicotine in cigarettes could therefore lead to reductions in smoking and, consequently, reduce the harms associated with nicotine use. Translating these empirical data into informed discussions of the potential impact of a nicotine reduction policy deserves further attention.

## Declarations of Interest

Author ECD received financial compensation from the World Health Organization to write this manuscript. The authors declare that they have no other known competing financial interests or personal relationships that could have appeared to influence the work reported in this paper.
